# Comparison of Gut Microbiome Profile of Chickens Infected with Three *Eimeria* Species Reveals New Insights on Pathogenicity of Avian Coccidia

**DOI:** 10.3390/microorganisms13122752

**Published:** 2025-12-03

**Authors:** Nianyu Xue, Dandan Liu, Qianqian Feng, Yu Zhu, Cheng Cheng, Feiyan Wang, Shijie Su, Jinjun Xu, Jianping Tao

**Affiliations:** 1 College of Veterinary Medicine, Yangzhou University, Yangzhou 225009, China; 2 Jiangsu Co-Innovation Center for Prevention and Control of Important Animal Infectious Diseases and Zoonoses, Yangzhou University, Yangzhou 225009, China

**Keywords:** *Eimeria*, gut microbiota, comparison, full-length 16S rRNA gene, chicken

## Abstract

Avian coccidiosis is an intestinal disease caused by *Eimeria* spp. infection. A deeper understanding of the interaction between host gut microbiota and the *Eimeria* parasite is crucial for developing alternative therapies to control avian coccidiosis. Here, we used full-length sequencing of 16S ribosomal RNA amplicons to compare changes in the gut microbiota of chickens infected with *Eimeria tenella*, *Eimeria maxima*, and *Eimeria necatrix*, aiming to identify both species-specific and common alterations in gut microbiota at 4 and 10 days post-infection. The result revealed that infection with all three *Eimeria* species led to a decrease in the abundance of the microbial genera *Limosilactobacillus*, *Streptococcus*, *Alistipes*, *Lactobacillus* and *Phocaeicola*, while the abundance of *Bacteroides*, *Escherichia* and *Ligilactobacillus* increased. *Escherichia* and *Enterococcus* were most abundant in the jejunum of the *E. necatrix*-infected group and in the cecum of the *E. tenella*-infected group, whereas *Megamonas* abundance was highest in the *E. maxima*-infected group. LEfSe analysis showed that infection with all three *Eimeria* species significantly reduced the abundance of 13 bacterial species, including *Acetilactobacillus jinshanensis*, *Bacteroides ndongoniae*, *Barnesiella viscericola*, *Christensenella minuta*, *Enterocloster clostridioformis, Gemella haemolysans*_A, *Granulicatella adiacens*, *Lawsonibacter* sp000177015, *Limosilactobacillus reuteri*, *Limosilactobacillus reuteri*_D, *Limosilactobacillus vaginalis*_A, *Limosilactobacillus caviae*, *Limosilactobacillus vaginalis*. Infection with *E. tenella* significantly increased the abundance of five bacterial species, including *Bacteroides fragilis*, *Enterococcus cecorum*, *Helicobacter pylori*, *Methylovirgula ligni*, and *Phocaeicola* sp900066445. Infection with *E. maxima* significantly increased the abundance of seven bacterial species, including *Clostridioides difficile*, *Faecalibacterium prausnitzii*, *Mediterraneibacter torques*, *Muribaculum intestinale*, *Mediterraneibacter massiliensis*, *Phascolarctobacterium faecium*, and *Phocaeicola plebeius*. Infection with *E. necatrix* significantly increased the abundance of seven bacterial species, including *Alistipes* sp900290115, *Anaerotignum faecicola*, *Bacteroides fragilis*_A, *Escherichia coli*, *Harryflintia acetispora*, *Pseudoclostridium thermosuccinogenes*, and *Tidjanibacter inops*_A. The results showed that *Eimeria* infection causes significant species- and time-dependent changes in the gut microbiota of chickens. These findings enhance our understanding of coccidiosis pathogenesis and offer potential targets for developing probiotics.

## 1. Introduction

Coccidiosis, caused by *Eimeria* species of the apicomplexan parasite, is a significant disease in chickens, impacting poultry production and resulting in approximately USD 10.36 billion in global losses annually [1]. Currently, coccidiosis control strategies rely heavily on chemoprophylaxis and, to a certain extent, live vaccines [2]. Unfortunately, the extensive use of drugs has inevitably led to the emergence of drug resistance, as well as the drug residues in the food chain and environment [3]. Therefore, it is necessary to adopt control strategies that replace antibiotics.

The gastrointestinal tract (GIT) in poultry harbors a diverse microbial community that serves a crucial role in digestion and protection [4]. Microbes located in the GIT mainly maintain homeostasis of the intestinal mucosa by digestion of food sources, providing the energy needed to induce the intestinal immune system to fight against aggressions of other microorganisms [5,6]. A standard or balanced gut microbiota reduces host susceptibility to pathogenic parasites like *Eimeria* spp. [7,8]. Studies have shown that the intestinal damage caused by *Eimeria* parasite colonization not only affects epithelial cells, but causes great disruption of gut microbial communities of chicken, promoting colonization and proliferation of other pathogens such as *Clostridium perfringens*, causing susceptibility of infected chickens to secondary diseases, thus increasing chicken mortality [9,10,11]. In addition, modulation of gut microbiota by probiotics protected early chicks against *Eimeria* infection [12,13,14]. Therefore, microbiota plays an important role in chickens’ resistance to coccidiosis and maintaining healthy growth and development.

Among the seven recognized species infecting chickens, *E. tenella*, *E. maxima*, and *E. necatrix* are frequently reported as economically important species, with their relative prevalence and significance varying according to production system (broilers, layers, or breeders), bird age, and geographic region [15,16,17]. *E. tenella* mainly infects the cecum of the host chicken, and *E. maxima* targets the jejunum of the small intestine, whereas *E. necatrix* is somewhat different from *E. tenella* and *E. maxima*, namely its first- and second-generation meronts located in the mid-intestinal area, and third-generation meronts and later gametogony only in the caecum [18,19,20,21]. Infection with these three *Eimeria* species disrupts intestinal integrity and microbiota balance, leading to impaired nutrient absorption, poor growth performance, increased mortality, and heightened vulnerability to secondary infections in chickens [22,23,24,25,26,27]. However, there are no reports on the similarities and differences in the impact of these three *Eimeria* species infections on the intestinal microbiota of chickens.

In this study, we used PacBio SMRT sequencing of full-length 16S rRNA amplicon to investigate infected chicken intestinal bacterial microbiota with *E. tenella*, *E. maxima*, and *E. necatrix*, respectively, to explore the effect of coccidia infection on gut microbiome diversity and composition in chickens, attempting to unveil the coccidian species-specific and common characteristics of changes in gut microbiota caused by different *Eimeria* spp. infection. The findings of this study may assist in selecting probiotics for the prevention and control of all three *Eimeria* species infections, as well as in understanding the correlation between microbiota changes and *Eimeria* infection, and in elucidating the pathogenicity of chicken coccidia.

## 2. Materials and Methods

### 2.1. Chickens and Parasites

One-day-old specific pathogen-free (SPF) White Leghorn chickens were obtained from Lihua Agricultural Technology Co., Ltd. (Yuyao, China) and raised under coccidia-free conditions in wire cages. Each cage housed 20 chickens, with each bird provided an individual floor area of 0.075 m^2^, ensuring adequate space for normal activity. The housing system was equipped with appropriate ventilation, temperature regulation, and sanitary management. Feed and water were supplied ad libitum. The chickens were provided a standard corn- and soybean-based diet purchased from Jiangsu Xietong Pharmaceutical Bio-Engineering Co., Ltd. (Nanjing, China). No antibiotics or anticoccidial agents were administered throughout the experiment. The Yangzhou strains of *E. tenella*, *E. maxima*, and *E. necatrix* used in this study were isolated by the Parasitology Research Laboratory at Yangzhou University, China, and confirmed through microscopic examination and sequence analysis of the internal transcribed spacer region of genomic DNA. These strains have been preserved in our laboratory [28,29,30].

### 2.2. Experimental Design

A total of 80 one-day-old SPF chickens were randomly divided into 4 groups (*n* = 20): the group infected with *E. tenella* (Et), the group infected with *E. maxima* (Em), the group infected with *E. necatrix* (En), and the unchallenged control group (Uc). At 21 days of age, the Et group was infected with 5 × 10^4^ *E. tenella* sporulated oocysts per chicken, the Em group with 5 × 10^4^ *E. maxima* sporulated oocysts per chicken, and the En group with 1.5 × 10^4^ *E. necatrix* sporulated oocysts per chicken. At 4 days post-infection (dpi) during the acute phase and at 10 dpi during the convalescent period, five chickens from each group were euthanized using CO_2_ inhalation followed by cervical dislocation. The intestines were dissected and visually inspected to identify the presence of any gross lesions. Following this, the intestinal mucosa was scraped, mounted on a microscope slide, and examined microscopically for parasites. Then the entire gastrointestinal tract was dissected from the chickens, and 200 mg of jejunum (the middle segment between the duodenal loop and Meckel’s diverticulum) and cecum contents were collected, respectively. The samples were stored at −80 °C. The sample information is shown in Table S1.

### 2.3. DNA Extraction, Library Construction, and Sequencing

Microbial DNA was extracted from the jejunum and cecum content of chickens using the E.Z.N.A.^®^ Soil DNA Kit (Omega Bio-tek, Norcross, GA, USA) according to manufacturer’s protocols, respectively. The V1–V9 region of the bacteria 16S ribosomal RNA gene were amplified by PCR (95 °C for 2 min, followed by 27 cycles at 95 °C for 30 s, 55 °C for 30 s, and 72 °C for 60 s and a final extension at 72 °C for 5 min) using primers 27F 5′-AGRGTTYGATYMTGGCTCAG-3′ and 1492R 5′-RGYTACCTTGTTACGACTT-3′. Amplicons were extracted from 2% agarose gels and purified using the AxyPrep DNA Gel Extraction Kit (Axygen Biosciences, Union City, CA, USA). The purified amplicon pool PCR products were used to construct PacBio SMRT sequencing libraries (Shanghai Biozeron Biotechnology Co., Ltd., Shanghai, China).

### 2.4. Bioinformatics and Data Analysis

PacBio raw reads were processed using the SMRT Link Analysis software version 9.0 to obtain demultiplexed circular consensus sequence (CCS) reads with the following settings: minimum number of passes = 3, minimum predicted accuracy = 0.99. Raw reads were processed through SMRT Portal to filter sequences for length (<800 or >2500 bp) and quality. Sequences were further filtered by removing barcode, primer sequences, chimeras and sequences if they contained 10 consecutive identical bases. OTUs were clustered with 98.65% similarity cutoff using UPARSE (version 7.1, http://drive5.com/uparse/, accessed on 30 November 2025) and chimeric sequences were identified and removed using UCHIME. The phylogenetic affiliation of each 16S rRNA gene sequence was analyzed by RDP Classifier (https://sourceforge.net/projects/rdp-classifier/, accessed on 30 November 2025) against the silva (SSU138)16S rRNA database using confidence threshold of 70% [31].

### 2.5. Statistical Analyses

Rarefaction analysis was performed using Mothur v.1.21.1 to calculate diversity indices, including Chao1 and Shannon indices [32]. Beta diversity was assessed using UniFrac, and principal component analysis (PCoA) results were generated using the community ecology package R-forge. The PCoA figure was produced using the Vegan package (version 2.6-2), and one-way permutational analysis of variance (PERMANOVA) was conducted [33,34]. Venn diagrams were generated using the online tool “Draw Venn Diagram” (http://bioinformatics.psb.ugent.be/webtools/Venn, accessed on 30 November 2025) to visualize overlapping and unique OTUs during the treatment process. The OTU data was filtered using the following steps: firstly, excluding “unclassified” and “norank” entries; secondly, removing OTUs with an abundance of less than one; finally, selecting core OTUs for plotting. The pheatmap package (version 1.0.12) was used to visualize the relationships through a correlation heatmap. All statistical analyses were conducted using the R stats package (version 4.0.2). Differences in species’ relative abundances were analyzed using the LEfSe (Linear Discriminant Analysis Effect Size) method [35]. The Phylogenetic Investigation of Communities by Reconstruction of Unobserved States (PICRUSt2), developed by [36] (available at http://picrust.github.io/picrust/tutorials/genome_prediction.html, accessed on 30 November 2025), was employed to predict changes in microbiota function across different samples using the Kyoto Encyclopedia of Genes and Genomes (KEGG) database. Comparisons between experimental groups were carried out using ANOVA followed by Tukey’s honest significant differences (HSD) post hoc test. *p*-values of <0.05 were considered significant.

## 3. Results

### 3.1. Clinical and Pathological Findings

Four days after infection with *E. tenella*, the chickens appeared depressed and exhibited diarrhea with a small amount of bright red blood. Necropsy of chickens showed that the cecum pouch became slightly enlarged, and some bleeding foci presented on the mucosal surface. Except for the presence of some blood, the cecal contents were normal. Microscopic examination of jejunal mucosal scrapings revealed no parasites (Figure S1A). In contrast, examination of scrapings from the lesioned areas of the cecum revealed numerous large second-generation schizonts (Figure S1B). At 10 dpi with *E. tenella,* the chickens appeared normal with no signs of diarrhea. The appearance of the cecum had returned to normal, but the cecal wall was thickened. Microscopic examination of jejunal mucosal scrapings revealed no parasites or oocysts (Figure S1C), whereas a large number of oocysts were observed in cecal mucosal scrapings (Figure S1D). No obvious lesions were observed in the jejunum at 4 and 10 dpi, respectively.

Four days after infection with *E. maxima*, the chickens exhibited ruffled feathers, depressed, and cotton thread-like feces. Necropsy revealed mild swelling of the small intestine and the presence of orange mucus in the intestinal lumen. Microscopic examination of the jejunal mucosal scrapings from areas with lesions revealed the presence of developing gametocytes (Figure S1E). In contrast, no parasites were found in the microscopic examination of cecal mucosal scrapings (Figure S1F). Ten days after infection with *E. maxima*, the chickens appeared normal, with no diarrhea. Necropsy revealed mild swelling of the jejunum, with scar tissue observed in the mucosa and a small amount of orange mucus in the intestinal lumen. Microscopic examination of jejunal mucosal scrapings revealed the presence of oocysts (Figure S1G), whereas no parasites or oocysts were found in the cecal mucosal scrapings (Figure S1H). No obvious lesions were observed in the cecum at 4 and 10 dpi, respectively.

Four days after infection with *E. necatrix*, the chickens appeared depressed and exhibited diarrhea with blood. The necropsy of chickens showed that mild swelling of the small intestine, and its serosal surface presented some small white plaques and red petechiae; the mucosa thickened; and the lumen filled with fluid, blood, and tissue debris. Microscopic examination of the jejunal scrapings from areas with lesions revealed the presence of large schizonts (Figure S1I). In contrast, no parasites were found in the cecal mucosal scrapings (Figure S1J). At 10 dpi with *E. necatrix*, the chickens appeared normal, with no diarrhea. Scar tissue was observed in the mucosa of the small intestine. Microscopic examination of the scrapings of the areas with lesion appeared a large number of large schizonts (Figure S1K). In addition, microscopic examination of cecal mucosal scrapings appeared the presence of oocysts (Figure S1L). No obvious lesions were observed in the cecum at 4 and 10 dpi, respectively.

### 3.2. Jejunum and Cecum 16S rRNA Sequences

After quality filtering and assembly, we obtained a total of 2,639,344 sequences, with an average sequence number of 32,775 and 33,208 sequences per sample for the jejunum and cecum, respectively. The average sequence lengths for jejunum and cecum were 1480 bp and 1461 bp, respectively (Table S2). Sequencing depth was sufficient for all samples, as confirmed by rarefaction and Shannon-Wiener curves (Figure S2).

### 3.3. Shared and Unique Core Microbial Populations

To investigate the microbial changes in the jejunum and cecum following infection with *E. tenella*, *E. maxima* and *E. necatrix*, we identified the unique and shared unique operational taxonomic units (OTUs) in the infected and control groups, with a total of 130,962 OTUs detected in 80 samples (Table S3), only the core OTUs were selected for visualization and further analysis.

**Figure 1:**
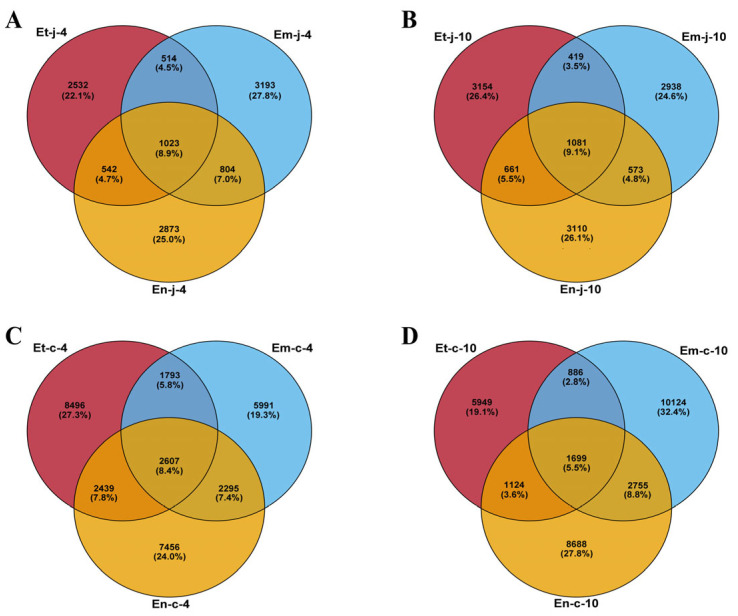
Venn diagram showing the number of unique and shared core OTUs in the microbial content of jejunum and cecum. Core OTUs of jejunum (**A**,**B**) at 4 and 10 dpi with *E. tenella*, *E. maxima*, *E. necatrix* group and control group. Core OTUs of cecum (**C**,**D**) at 4 and 10 dpi with *E. tenella*, *E. maxima*, *E. necatrix* group and control group.

In the jejunum: Four days after infection with *E. tenella, E. maxima* and *E. necatrix*, a total of 2532, 3193 and 2873 unique OTUs were detected, respectively, with 1023 OTUs shared among the three infected groups (Figure 1A). Ten days after infection, 3154, 2938 and 3110 unique OTUs were detected for *E. tenella, E. maxima* and *E. necatrix*, respectively, with 1081 OTUs shared among the three infected groups (Figure 1B).

In the cecum: Four days after infection with *E. tenella, E. maxima* and *E. necatrix*, a total of 8496, 5991 and 7456 unique OTUs were detected, respectively, with 2607 OTUs shared among the three infected groups (Figure 1C). Ten days after infection with *E. tenella, E. maxima* and *E. necatrix*, a total of 5949, 10124 and 8688 unique OTUs were detected, respectively, with 1699 OTUs shared among the three infected groups (Figure 1D).

### 3.4. Alpha and Beta Diversity of Jejunal and Cecal Microbial Constitution After E. tenella, E. maxima and E. necatrix Infection

**Figure 2:**
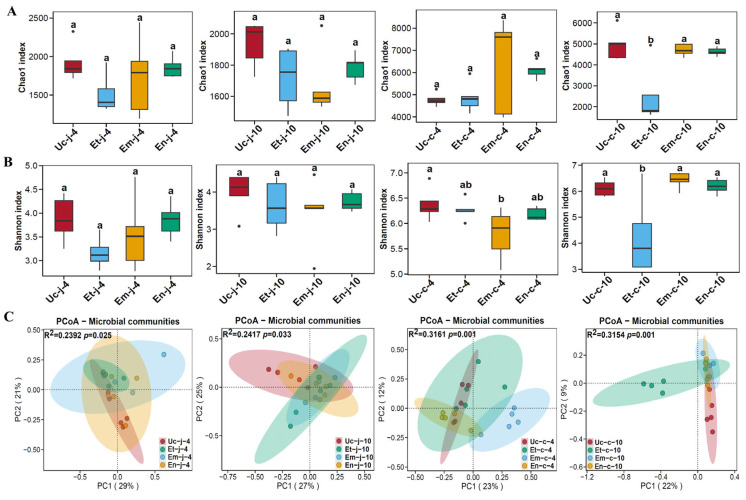
Alpha diversity and Principal coordinate analysis between different groups. (**A**) Chao1 index, (**B**) Shannon index. (**C**) Principal coordinate analysis between 4 groups. Different letters on the bar graph represent significant differences between groups (*p* < 0.05).

Alpha diversity was assessed using the Chao 1 index for microbial richness and the Shannon index for species diversity (Table S4). The Chao 1 index indicated that infections with these three *Eimeria* species at 4 and 10 dpi led to a decrease in jejunal microbial species richness (*p* > 0.05, Figure 2A). In the cecum, *E. tenella* infection at 10 dpi resulted in a significant decrease in species richness, while the groups infected with *E. maxima* or *E. necatrix* only showed a slight decrease (*p* < 0.05, Figure 2A). The Shannon index showed that species diversity in the jejunum decreased in all infected groups at 4 and 10 dpi, respectively (*p* > 0.05, Figure 2B). In the cecum, the species diversity significantly decreased after 4 days of infection with *E. maxima*, while a significant decrease was observed after 10 days of infection with *E. tenella* (*p* < 0.05, Figure 2B).

Principal Coordinate Analysis (PCoA) using the Bray–Curtis distance metric was conducted to explore differences in gut microbiota between groups. In the jejunum, at 4 dpi with *E. tenella*, *E. maxima*, and *E. necatrix* infection, the microbial structures changed significantly, and the communities separated between groups. PC1 and PC2 explained variances of 29% and 21%, respectively. PERMANOVA analysis revealed significant differences (R^2^ = 0.2392, *p* = 0.025). After 10 days of infection, the microbial communities remained distinct among groups, with PC1 and PC2 explaining variances of 27% and 25%, respectively. PERMANOVA analysis again showed significant differences (R^2^ = 0.2417, *p* = 0.033) (Figure 2C).

In the cecum, at 4 dpi with *E. maxima* infection, the microbial community was significantly distinct from those infected with *E. tenella* and *E. necatrix*, with PC1 and PC2 explaining 23% and 12% of the variance, respectively. PERMANOVA analysis revealed significant differences between the groups (R^2^ = 0.3161, *p* = 0.001). At 10 dpi with *E. tenella* infection, the microbial community was significantly different from those infected with *E. maxima* and *E. necatrix*, with PC1 and PC2 explaining 22% and 9% of the variance, respectively. PERMANOVA analysis again showed significant differences between the groups (R^2^ = 0.3154, *p* = 0.001) (Figure 2C).

### 3.5. Bacterial Taxa in the Jejunum and Cecum After E. tenella, E. maxima and E. necatrix Infection

The taxonomic richness of 80 samples varied across different taxonomic levels, leading to the identification of 31 phyla, 60 classes, 123 orders, 260 families, 756 genera, and 1918 species (Table S5).

#### 3.5.1. At the Phylum Level

**Figure 3:**
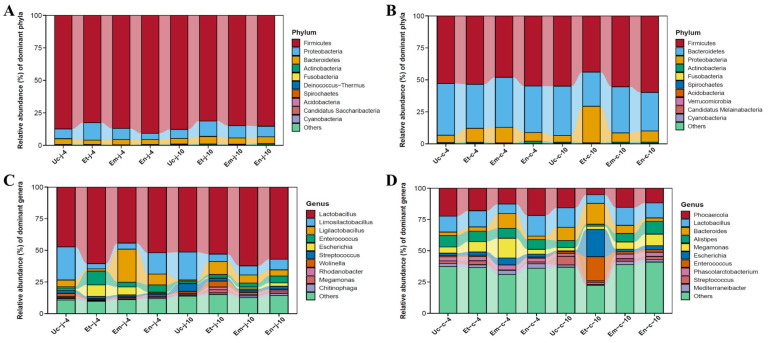
The gut microbiota structure of the jejunum and cecum at the phylum (**A**,**B**) and Genus (**C**,**D**) level of at 4 and 10 dpi with *E. tenella*, *E. maxima* and *E. necatrix* and control groups.

In the jejunum: Firmicutes, Proteobacteria, and Bacteroidetes were the most abundant phyla among the top ten at both 4-day and 10-day post-infection groups, as well as the control group (Figure 3A). At 4 dpi with all three *Eimeria* species, the abundance of Bacteroidetes decreased. The abundance of Firmicutes decreased after infection with *E. tenella* and *E. maxima*, respectively, but increased after infection with *E. necatrix*. The abundance of Proteobacteria increased after infection with *E. tenella* and *E. maxima* but decreased after infection with *E. necatrix*. At 10 dpi with all three *Eimeria* species, the abundance of Firmicutes decreased, while the abundance of Proteobacteria and Bacteroidetes increased (Figure 3A).

In the cecum: Firmicutes, Bacteroidetes, and Proteobacteria were the most abundant phyla among the top ten at both 4-day and 10-day post-infection groups, as well as the control group (Figure 3B). At 4 dpi with all three *Eimeria* species, the Bacteroidetes abundance decreased, while the Proteobacteria abundance increased. The Firmicutes abundance increased after infection with *E. tenella* and *E. necatrix* but decreased after infection with *E. maxima*. At 10 dpi with all three *Eimeria* species, the Bacteroidetes abundance continued to decrease, while that of Proteobacteria increased. The abundance of Firmicutes increased following infection with *E. maxima* and *E. necatrix* but decreased after infection with *E. tenella* (Figure 3B).

#### 3.5.2. At the Genus Level

In the jejunum: At 4 dpi with all three *Eimeria* species, the *Limosilactobacillus* abundance decreased (Figure 3C). At 10 dpi with all three *Eimeria* species, the abundance of *Limosilactobacillus* and *Streptococcus* decreased, while the *Ligilactobacillus* abundance increased. The abundance of *Escherichia* and *Enterococcus* were higher in the group infected with *E. necatrix* compared to the other two infected groups (Figure 3C).

In the cecum: At 4 dpi with all three *Eimeria* species, the *Alistipes* abundance decreased, while the abundance of *Bacteroides* and *Escherichia* increased. The *Megamonas* abundance was higher in the group infected with *E. maxima* than in the other two infected groups (Figure 3D). At 10 dpi with all three *Eimeria* species, the abundance of *Lactobacillus* and *Phocaeicola* decreased. The abundance of *Escherichia* and *Enterococcus* were higher in the group infected with *E. tenella* compared to the other two *Eimeria* infected groups. In contrast, the abundance of *Alistipes* and *Megamonas* increased in the groups infected with *E. maxima* and *E. necatrix* (Figure 3D).

#### 3.5.3. At the Species Level

**Figure 4:**
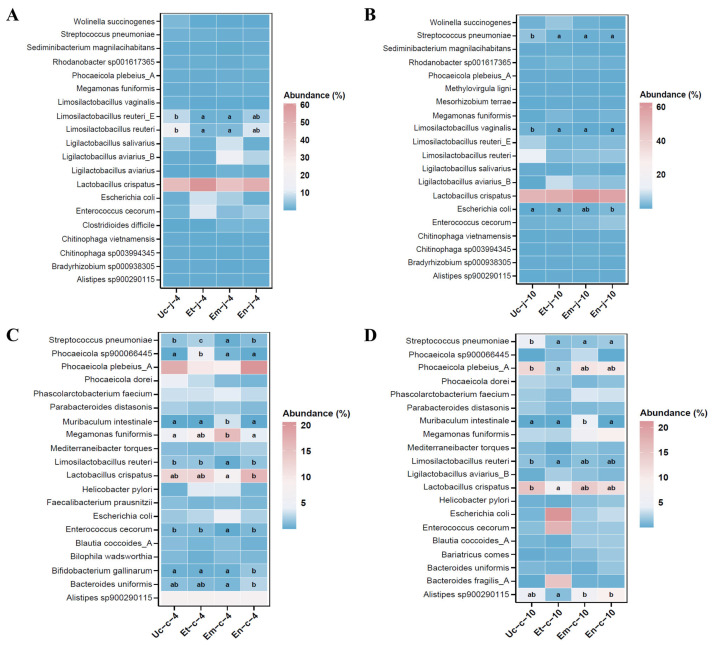
Significant differences in the abundance of the top 20 species in the jejunum (**A**,**B**) and cecum (**C**,**D**) microbiota at 4 and 10 dpi with *E. tenella*, *E. maxima*, and *E. necatrix*. Different letters on the bar graph represent significant differences between groups (*p* < 0.05).

To identify the key microbiota involved in the process of *E. tenella*, *E. maxima* and *E. necatrix* infection in chickens, we performed differential abundance analysis of the jejunal and cecal microbiota between the different groups. The results for the top 20 species with significant differences are shown in Figure 4.

In the jejunum: At 4 dpi with all three *Eimeria* species, the abundance of *Limosilactobacillus vaginalis*, *Streptococcus pneumoniae*, *Limosilactobacillus reuteri* and *Limosilactobacillus reuteri*_E decreased, with the latter two bacteria showing a significant reduction in the groups infected with *E. tenella* and *E. maxima* (*p <* 0.05; Figure 4A). In contrast, the abundance of *Enterococcus cecorum* increased. The abundance of *Megamonas funiformis* and *Ligilactobacillus aviarius*_B decreased in the group infected with *E. tenella* but increased in the groups infected with *E. maxima* and *E. necatrix*. At 10 dpi with all three *Eimeria* species, the abundance of *Limosilactobacillus reuteri*, *Limosilactobacillus reuteri*_E, *Limosilactobacillus vaginalis* and *Streptococcus pneumoniae* decreased, with the latter two bacteria showing a significant reduction. The abundance of *Escherichia coli* significantly increased in the group infected with *E. necatrix* and was higher than that in the other two infected groups (*p <* 0.05; Figure 4B).

In the cecum: At 4 dpi, the abundance of *Streptococcus pneumoniae*, *Phocaeicola* sp900066445, and *Megamonas funiformis* significantly increased in the group infected with *E. tenella*. In contrast, in the group infected with *E. maxima*, the abundance of *Limosilactobacillus reuteri* and *Lactobacillus crispatus* significantly decreased, while that of *Muribaculum intestinale* significantly increased (*p <* 0.05; Figure 4C). At 10 dpi, the abundance of *Muribaculum intestinale* still significantly increased in the group infected with *E. maxima*. Conversely, *Limosilactobacillus reuteri* and *Lactobacillus crispatus* significantly decreased in the group infected with *E. tenella* (*p <* 0.05; Figure 4D).

### 3.6. LEfSe Analysis of Differences in the Microbiota of the Jejunum and Cecum After E. tenella, E. maxima and E. necatrix Infection

**Figure 5:**
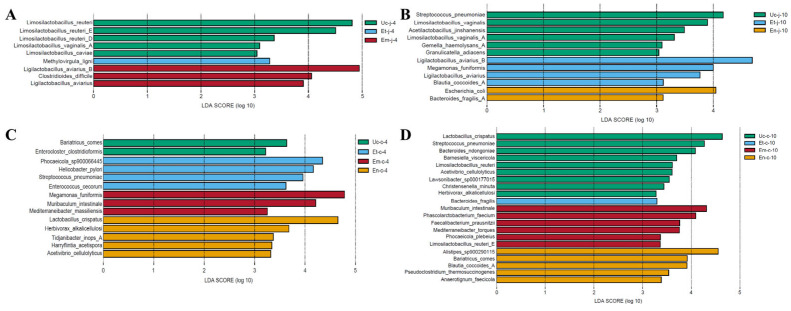
Differential enrichment of jejunal and cecal bacteria in response to *E. tenella*, *E. maxima*, and *E. necatrix* infections was assessed using LEfSe analysis. The top 50 jejunal (**A**,**B**) and cecal (**C**,**D**) species were compared between the three infected groups and the control group at 4 and 10 dpi. A significance threshold of *p* < 0.05 and an LDA score ≥ 3.0 were applied.

To identify groups exhibiting significant differences in bacteria species, we performed LEfSe analysis on the top 50 species in both the jejunum and cecum at the same timepoint among the three infection groups and the control group, with an LDA threshold set to ≥3.0. A total of 42 bacteria species showed significant differences in abundance among groups (Figure 5). Among these 42 bacteria species, the abundance of 13 species decreased across all three infection groups, while five species increased exclusively in the *E. tenella*-infected group, seven species in the *E. maxima*-infected group, and another seven species in the *E. necatrix*-infected group. Additionally, the abundance of four bacteria species increased in infection group. The details of the results were summarized as follows.

In the jejunum: At 4 dpi with all three *Eimeria* species, the abundance of *Limosilactobacillus reuteri*, *Limosilactobacillus reuteri*_D, *Limosilactobacillus vaginalis*_A, and *Limosilactobacillus caviae* decreased significantly (Figure 5A). The abundance of *Methylovirgula ligni* increased in the *E. tenella*-infected group, whereas *Clostridioides difficile* increased significantly in the *E. maxima*-infected group (Figure 5A). At 10 dpi with all three *Eimeria* species, the abundance of *Limosilactobacillus vaginalis*, *Acetilactobacillus jinshanensis*, *Limosilactobacillus vaginalis*_A, *Gemella haemolysans*_A, and *Granulicatella adiacens* decreased significantly (Figure 5B). Although *Megamonas funiformis*, *Ligilactobacillus aviarius*, *Ligilactobacillus aviarius*_B and *Blautia coccoides*_A increased in the *E. tenella*-infected group, none of them belong to species-dependent changes. *Escherichia coli* and *Bacteroides fragilis*_A increased in the *E. necatrix*-infected group (Figure 5B).

In the cecum: At 4 dpi with all three *Eimeria* species, the abundance of *Enterocloster clostridioformis* decreased significantly. *Phocaeicola* sp900066445, *Helicobacter pylori*, and *Enterococcus cecorum* increased significantly in the *E. tenella*-infected group, *Muribaculum intestinale* and *Mediterraneibacter massiliensis* in the *E. maxima*-infected group, and *Tidjanibacter inpos*_A and *Harryflintia acetispora* in the *E. necatrix*-infected group (Figure 5C). At 10 dpi with all three *Eimeria* species, the abundance of *Bacteroides ndongoniae*, *Barnesiella viscericola*, *Limosilactobacillus reuteri*, *Lawsonibacter* sp000177015, *Christensenella minuta*, *Acetivibrio cellulolyticus*, *Lactobacillus crispatus*, *Streptococcus pneumoniae*, and *Herbivorax alkalicellulosi* decreased significantly, but the last four species belong to no common changes caused by *Eimeria* infection. *Bacteroides fragilis* increased significantly in the *E. tenella*-infected group, *Muribaculum intestinale*, *Phascolarctobacterium faecium*, *Faecalibacterium prausnitzii*, *Mediterraneibacter torques, Phocaeicola plebeius*, and *Limosilactobacillus reuteri*_E in the *E. maxima*-infected group, and *Alistipes* sp900290115, *Pseudoclostridium thermosuccinogenes*, and *Anaerotignum faecicola* in the *E. necatrix*-infected group (Figure 5D).

### 3.7. Functional Prediction of Microbiota

**Figure 6:**
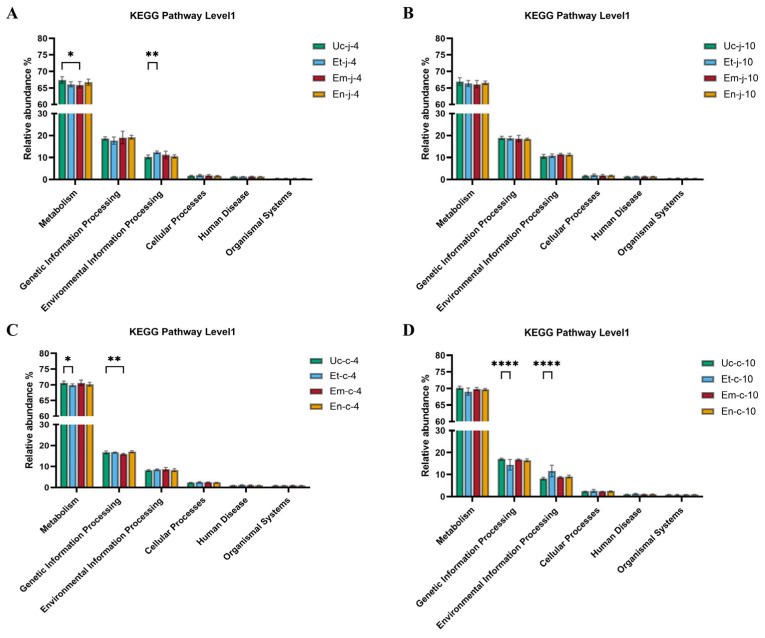
Functional prediction analysis of microbiota in jejunal (**A**,**B**) and cecal (**C**,**D**) contents of chickens. *, *p* < 0.05, **, *p* < 0.01, and ****, *p* < 0.0001.

Change in microbial composition and structure are closely related to functional alteration of microbes. Thus, KEGG analysis was further used to predict the altered pathways in our study. At 4 dpi in the jejunum, distinct microbial functional changes were observed between species: metabolism-related functions were significantly reduced in the *E. maxima*-infected group compared to the control (*p* < 0.05), while the *E. tenella*-infected group showed a significant increase in environmental information processing functions (*p* < 0.01; Figure 6A). By 10 dpi, no significant differences in predicted jejunal microbial functions were found between infected groups and the control (*p* > 0.05; Figure 6B).

Cecal microbial functional analysis further confirmed the differential impact patterns of different *Eimeria* species on microbial community functions. At 4 dpi, the *E. tenella*-infected group hand a significantly lower relative abundance of predicted metabolic pathways compared to the control group (*p* < 0.05). In contrast, the *E. maxima*-infected group showed a significant reduction in genetic information processing functions (*p* < 0.01; Figure 6C). By 10 dpi, the *E. tenella*-infected group continued to exhibit low levels of genetic information processing functions, while environmental information processing functions increased significantly compared to the control group (*p* < 0.0001; Figure 6D).

## 4. Discussion

The enteric microflora plays a crucial role in the health, welfare, and productivity of commercially reared chickens. A balanced microbial community is essential for chickens to effectively utilize end-products of metabolic processes and to facilitate interactions between the host and diet [37]. However, this balance can be easily affected by various factors, such as *Eimeria* parasite infection [8,38]. Each *Eimeria* species presents a distinct pathognomonic profile and affects different sections of the intestine [39,40]. Consequently, the impact of *Eimeria* infection on the gut microbiota of chickens may vary depending on the specific *Eimeria* species. In addition, the gut microbiota of chickens is influenced by various factors, including age, sex, breed, diet, litter type and conditions, and the use of antimicrobials [41,42,43]. In this study, therefore, chickens were infected with *E. tenella*, *E. maxima*, and *E. necatrix* under identical experimental conditions to examine the composition and integrity of their gut microbiota.

In a well-balanced chicken gastrointestinal tract, the predominant bacterial groups are Firmicutes, Tenericutes, Bacteroidetes, Proteobacteria, and Actinobacteria [42,43,44]. In this study, at two timepoints, the predominant bacteria in the jejunum of both the *Eimeria*-infected and control groups were Firmicutes, Proteobacteria, and Bacteroidetes, whereas in the cecum, the predominant bacteria were Firmicutes, Bacteroidetes, and Proteobacteria. Compared to the control group, the abundance changes in Bacteroidetes and Proteobacteria showed a consistent pattern across the three *Eimeria* species. The abundance of Bacteroidetes decreased in both the jejunum and cecum, except for an increase in the jejunum at 4 dpi. Conversely, the abundance of Proteobacteria increased in both the jejunum and cecum, except for a decrease in the jejunum at 4 dpi with *E. necatrix*. However, the changes in Firmicutes abundance due to infections with the three species were inconsistent. Infections with *E. tenella* and *E. maxima* resulted in a reduction in Firmicutes abundance, except for an increase in the cecum at 4 dpi with *E. tenella* and at 10 dpi with *E. maxima*. Conversely, infection with *E. necatrix* led to an increase in Firmicutes abundance, except for a reduction in the jejunum at 10 dpi. Bacteroidetes are Gram-negative bacteria that inhabit various regions of the intestinal tract [42,45]. Fan et al. demonstrated that one week-old broiler chickens with high *Bacteroides* abundance exhibited significantly increased concentrations of short-chain fatty acids, particularly acetate, propionate, butyrate, and valerate in the cecum, which was associated with enhanced polysaccharide degradation capacity. Furthermore, these chickens showed increased expression of the tight-junction protein *claudin-1*, decreased expression of the pro-inflammatory cytokine *IL-1β*, and elevated expression of the anti-inflammatory cytokine *IL-10* in cecal tissues, indicating improved intestinal barrier function and reduced gut inflammation [46]. Proteobacteria contain a wide variety of remarkable conditional pathogens, such as some species of these genera (*Comamonas*, *Acinetobacter*, *Brucella*, *Shigella*, and *Escherichia*) [47]. The abundance of Proteobacteria has been regarded as a signature of dysbiosis and disease in humans [48]. The increased relative abundance of Proteobacteria leads to disease development and reduces chicken growth performance [49]. The increase in Proteobacteria has been widely documented across multiple *Eimeria* infection studies [50,51], suggesting this represents a common host response to enteric coccidiosis, although the magnitude and timing of changes may vary depending on the specific *Eimeria* species and intestinal segment examined. Firmicutes are mainly represented by the genera *Enterococcus*, *Ruminococcus*, *Clostridium*, *Lactobacillus*, *Faecalibacterium*, *Roseburia*, and *Eubacterium*. Members of Firmicutes can inhibit the growth of opportunistic pathogens and degrade complex carbohydrates. Other members, such as *Enterococcus* spp. and *Streptococcus* spp., typically occur in low abundance but may become pathogenic during intestinal dysbiosis [47,52]. The results suggest that *Eimeria* infection disrupts the gut microbial communities of chicken and increases the risk of secondary infections from opportunistic pathogens. Variations in the abundance of Firmicutes among the three *Eimeria* species may reflect differences in their colonization sites and pathogenic characteristics.

The chicken gastrointestinal tract harbor diverse communities of commensal, symbiotic and pathogenic microorganisms [6,8,53]. *Eimeria* infection can reduce the abundance of commensal and symbiotic bacteria while increasing the abundance of pathogenic bacteria [13,22,54,55,56,57]. In the current study, we observed similar results. Our results showed that the abundance changes in *Limosilactobacillus*, *Enterococcus*, *Streptococcus*, *Escherichia*, and *Ligilactobacillus* due to *Eimeria* parasite infection were consistent across *E. tenella*, *E. maxima*, and *E. necatrix*. Specifically, *Limosilactobacillus* abundance decreased in both the jejunum and cecum of chickens at 4 and 10 dpi. Consistent with previous studies, a similar decrease in *Limosilactobacillus* abundance was reported in the ileum of chickens following *E. maxima* infection [52], indicating that this response may occur consistently across different *Eimeria* species and intestinal locations. *Enterococcus* abundance increased while *Streptococcus* abundance decreased, except in the cecum at 4 dpi, where both abundances were extremely low. *Escherichia* abundance increased, except for a decrease in the jejunum at 4 dpi with *E. necatrix*. *Ligilactobacillus* abundance was extremely low in the cecum but increased in the jejunum, except for a decrease at 4 dpi with *E. tenella*. *Limosilactobacillus* and *Ligilactobacillus* are two newly recognized genera that originated from *Lactobacillus* and belong to the *Lactobacillaceae* family [58,59]. Nii et al. reported that *Limosilactobacillus reuteri* treatment increased ileal villus height and enhanced mucosal barrier function against *Salmonella Typhimurium* challenge in broiler chicks [60]. Additionally, dietary supplementation with *Limosilactobacillus reuteri* significantly improved body weight, average daily gain, and feed conversion ratio in broiler chickens [61]. He et al. demonstrated that *Ligilactobacillus salivarius* XP132 significantly reduced *Salmonella Pullorum* in liver, spleen, intestinal contents, and eggs, effectively preventing both horizontal and vertical transmission. This protective effect was associated with enhanced immune responses, including upregulated *IFN-γ* and downregulated pro-inflammatory (*IL-1β, IL-6*, *IL-8*, and *TNF-α*) [62]. Some *Streptococcus* species are commensal. Certain strains isolated from chicken ceca in previous studies have demonstrated either butyrate production [63] or probiotic potential [64]. *Enterococcus* and *Escherichia* include opportunistic pathogens, and their elevation suggests that *Eimeria* infection may promote the proliferation of potentially harmful bacteria in the jejunum, increasing the risk of secondary infections or intestinal inflammation [47,52,65].

Furthermore, our results revealed no consistent pattern in the abundance changes in *Alistipes, Phocaeicola*, *Bacteroides*, and *Megamonas* due to infection with the three *Eimeria* species in the cecum. In the jejunum, their abundance was very low, except for an increase in *Megamonas* at 10 dpi. Additionally, infections with *E. tenella* and *E. necatrix* resulted in an increase in *Lactobacillus*, except for a reduction in the cecum at 10 dpi. In contrast, infection with *E. maxima* led to a reduction in *Lactobacillus* abundance, except for an increase in the jejunum at 10 dpi. *Alistipes* is a genus member of the family *Rikenellaceae*. *Alistipes* dysbiosis can be either beneficial, or harmful [66]. *Bacteroides* and *Phocaeicola*, members of the family *Bacteroidaceae*, are of clinical importance in human or veterinary medicine due to their presence in gut microbiota [67]. Members of both genera are significant gut commensals that can degrade and ferment mucin or complex polysaccharides from plants. Outside the intestinal tract, however, *Bacteroides* spp. and *Phocaeicola* spp. may participate in various pathogenic processes [45,68]. *Megamonas*, a genus within the Firmicutes, was previously regarded as a ‘biomarker’ of diet and lifestyle in humans [69]. Previous research showed that *Megamonas* functions as a hydrogen sink in the cecum of broiler chickens by enhancing the production of short-chain fatty acids [70]. Additionally, Niu et al. found that *Megamonas* is positively correlated with reproductive performance of hens and has lower abundance in *Salmonella pullorum*-positive hens [71]. Together, these results indicate that *Eimeria* infection causes distinct microbial changes at 4 dpi during the acute phase and at 10 dpi during the convalescent period. Beneficial bacteria, such as *Limosilactobacillus* and *Lactobacillus*, are generally suppressed, whereas opportunistic pathogens like *Escherichia* and *Enterococcus*, or compensatory genera like *Ligilactobacillus* and *Megamonas*, exhibit species- or site-specific alterations. Specifically, *E. tenella* in the cecum and *E. necatrix* in the jejunum more strongly promote pathogenic bacterial overgrowth within their respective intestinal sections. In contrast, *E. maxima* may induce unique metabolic adaptations, such as an increase in *Megamonas*. These differences are likely related to the distinct pathogenic mechanisms and tissue-specific effects of each *Eimeria* species.

LEfSe analysis revealed that infection with all three *Eimeria* species significantly decreased the abundance of 13 bacterial species. Out of 13 bacterial species, *Acetilactobacillus jinshanensis*, *Limosilactobacillus reuteri*, *Limosilactobacillus reuteri*_D, *Limosilactobacillus vaginalis*, *Limosilactobacillus vaginalis*_A, and *Limosilactobacillus caviae* are lactic acid bacteria crucial for the gut microbiota [58,59]. These bacteria help maintain an acidic intestinal environment, inhibit pathogen colonization, and regulate immunity [72,73,74]. A decrease in their abundance may weaken the intestinal barrier function [75]. The remaining seven bacterial species, including *Bacteroides ndongoniae*, *Barnesiella viscericola*, *Christensenella minuta*, *Enterocloster clostridioformis*, *Gemella haemolysans*_A, *Granulicatella adiacens*, and *Lawsonibacter* sp000177015, are either symbiotic or opportunistic pathogens [42,45,68,76,77]. *Christensenella minuta* was designated as the first member of the new *Christensenellaceae* family in the Firmicutes phylum [78]. Previous research showed that *Christensenellaceae* and *Christensenella munita* specifically can play a crucial role in maintaining a healthy gut microbiome [79]. *Gemella haemolysans* and *Granulicatella adiacens* are opportunistic pathogens [80,81]. Some *Gemella* species are known to infrequently cause systemic illnesses and are a component of the oral microbiome in humans [82]. *Granulicatella adiacens* is part of the normal commensal flora of human mouth, genital, and intestinal tracts, and rarely causes disease [81]. Previous research found that *Granulicatella* is significantly enriched in chickens with bacterial chondronecrosis and osteomyelitis as compared to healthy chickens [83]. *Enterocloster clostridioformis* is the reclassified name for *Clostridium clostridioforme* [84]. A study revealed that *Enterocloster clostridioformis* increases regulatory T cells in the mucosal immune system, which helps reduce the pathological damage caused by *Salmonella Typhimurium* infection [85]. However, clinical reports also documented cases of bacteremia caused by *Enterocloster clostridioformis* [86]. *Bacteroides ndongoniae* is a Gram-negative, non-spore-forming and non-motile bacillus [87]. *Barnesiella viscericola* is a member of the family *Porphyromonadaceae* isolated from chicken caecum [88]. *Lawsonibacter* sp000177015, belonging to the family *Ruminococcaceae*, is a bacterial species identified in the human gut, particularly in the context of knee synovitis [77]. The reduction in these symbiotic bacterial species may disrupt the intestinal microbiome balance and increase the risk of infection by other pathogens.

LEfSe analysis reveals that changes in bacterial species caused by different *Eimeria* species also exhibit species-specific characteristics. *E. tenella* infection significantly increased the abundance of *Bacteroides fragilis*, *Enterococcus cecorum*, *Phocaeicola* sp900066445, *Helicobacter pylori*, and *Methylovirgula ligni*. *E. maxima* infection significantly increased the abundance of *Clostridioides difficile*, *Faecalibacterium prausnitzii*, *Mediterraneibacter torques*, *Mediterraneibacter massiliensis*, *Muribaculum intestinale*, *Phascolarctobacterium faecium*, and *Phocaeicola plebeius*. *E. necatrix* infection significantly increased the abundance of *Escherichia coli*, *Alistipes* sp900290115, *Bacteroides fragilis*_A, *Anaerotignum faecicola, Harryflintia acetispora*, *Pseudoclostridium thermosuccinogenes*, and *Tidjanibacter inops*_A. Among these bacterial species, some species (such as *Enterococcus cecorum* and *Bacteroides fragilis*) are typically found in chickens as part of normal flora, whereas another species (such as *Mediterraneibacter massiliensis* and *Methylovirgula ligni*) were originally isolated from human samples or environmental sources.

Among five bacterial species associated with *E. tenella* infection, *Enterococcus cecorum* is a commensal bacteria and opportunistic pathogen that can cause outbreaks of Enterococcal spondylitis, commonly known as ‘kinky back’, in poultry [89]. A previous study reported that *Enterococcus* abundance increased over time in response to *E. tenella* infection [13]. *Bacteroides fragilis* is the most common opportunistic anaerobic pathogen [90]. It also is short-chain fatty acids (SCFAs)-producing bacteria [91]. Studies showed that high levels of *Bacteroides fragilis* in young chickens are associated with a more beneficial gut microbiome composition, potentially reducing inflammation [92,93]. *Phocaeicola* sp900066445 is a bacterial strain belonging to the *Bacteroidaceae* family. Previous research revealed that certain *Phocaeicola* species, like *Phocaeicola barnesiae*, may play a role in maintaining a healthy gut environment in chickens [67]. *Helicobacter pylori* is a flagellated pathogen that colonizes the human gastroduodenal mucosa and produces inflammation [94] and can be present in raw chicken meat. Additionally, studies have shown that this bacterium can survive in the gastrointestinal tract of chickens and can be present in their feces [95]. *Methylovirgula ligni* is an environmental bacterium [96]. Taken together, *E. tenella* infection promoted the proliferation of opportunistic pathogens and inflammation-associated bacteria, which exacerbated intestinal inflammation and damage.

Among seven bacterial species associated with *E. maxima* infection, *Clostridioides difficile* is a major opportunistic pathogen capable of producing potent cytotoxins (TcdA and TcdB) that can cause severe colitis [97]. *Clostridioides difficile* is associated with antibiotic-associated diarrhea and pseudomembranous colitis in humans [98], although there is no evidence that *Clostridioides difficile* is a relevant pathogen in poultry [99]. Sokol et al. demonstrated that secreted metabolites of *Faecalibacterium prausnitzii* blocked *NF-κB* activation and *IL-8* secretion in intestinal epithelial cells, reduced pro-inflammatory cytokine production, and promoted *IL-10* secretion [100]. Lenoir et al. further confirmed that butyrate was the key mediator of these anti-inflammatory effects [101]. These immunomodulatory properties contribute to improved intestinal barrier function and reduced inflammation. *Mediterraneibacter torque* and *Mediterraneibacter massiliensis* belong to the genus *Mediterraneibacter* within the family *Lachnospiraceae*, primarily found in the cecal microbiota of chickens [102]. *Mediterraneibacter torque*, previously classified under *Ruminococcus* species in *Clostridium* cluster XIVa [102], is known for its ability to degrade gastrointestinal mucin [103]. Studies have found that the *Ruminococcus_torques_group* may be the key gut microbiota contributing to the alterations of tracheal microbiota composition [104]. *Muribaculum intestinale*, a Gram-negative obligate anaerobe from the *Muribaculaceae* family, was first identified in the mouse gut microbiome. It has been linked to inflammatory bowel disease in both mice and human studies by several research groups [105]. A currently research revealed that *Muribaculum intestinale* limits *Salmonella Typhimurium* colonization by converting succinate to propionate in mice [106]. Therefore, following *E. maxima* infection, the increase in *Muribaculum intestinale* abundance in the cecum may trigger the production of pro-inflammatory factors and induce local inflammation. *Phascolarctobacterium faecium*, a bacterium commonly found in the human gastrointestinal tract within the family *Acidaminococcaceae*, is known for its ability to metabolize succinate and produce acetate and propionate. Research has indicated that *Phascolarctobacterium faecium* can help reverse the inflammatory phenotype associated with obesity [107]. *Phocaeicola plebeius*, formerly *Bacteroides plebeius*, is strictly anaerobic, Gram-negative, and non-spore forming bacterium [108]. Previous research has found that *Bacteroides* (*Phocaeicola*) *plebeius* can restructure the gut microbial community and produce beneficial metabolites, which inhibit the development of colitis-associated colon cancer [109]. Additionally, this species was identified as a potential host immunomodulator during *Salmonella* infection in chickens, playing a protective role [110]. In summary, *E. maxima* infection resulted in an imbalance between pathogenic and symbiotic bacteria, potentially leading to pseudomembranous enteritis and mucus layer loss, as well as triggering compensatory proliferation of anti-inflammatory bacteria.

Among seven bacterial species associated with *E. necatrix*, *Escherichia coli* is a bacterium crucial for maintaining intestinal health in both humans and animals. While most *Escherichia coli* strains are non-pathogenic, about 10–15% of intestinal coliforms consist of opportunistic and pathogenic serotypes that can cause infections in immunocompromised hosts, such as poultry [111]. Chen et al. [51] found that high-dose *E. necatrix* in chickens leads to increased abundance of *Escherichia coli*. *Alistipes* sp900290115 is isolated from various abscesses and may play an opportunistic pathogenic role in human diseases [112]. *Bacteroides fragilis*_A, a subtype of *Bacteroides fragilis*, may play a role in intestinal inflammation. *Anaerotignum faecicola* is a newly identified species within the genus *Anaerotignum*, isolated from human feces and belonging to the family *Lachnospiraceae* [113]. Studies have shown that *Anaerotignum* can produce acetate, propionate, and butyrate to provide energy to the host [114], and the abundance of *Anaerotignum* significantly increases in diarrhea mice with deficiency kidney-yang syndrome [115]. *Harryflintia acetispora*, isolated from chicken’s caecum, is characterized as a Gram-negative, curved rod-shaped bacterium capable of forming endospores [116]. Its exact role in the chicken gut and its potential impact on chicken health are still under investigation. *Pseudoclostridium thermosuccinogenes*, formerly *Clostridium thermosuccinogenes* [117], is a thermotolerant succinic acid-producing bacterium [118]. *Tidjanibacter* is a new genus derived from the *Alistipes* genus [119]. A recent study found that the abundance of *Tidjanibacter inops*_A has a positive correlation with the average daily weight gain of chickens and a negative correlation with serum IL-6 levels [120]. Collectively, *E. necatrix* infection triggered the activation of opportunistic pathogens, and led to metabolic bacterial disorders and proliferation, which may potentially exacerbate intestinal inflammation and metabolic imbalance.

Furthermore, the functional prediction analysis indicated that infection with three *Eimeria* species significantly altered the host’s intestinal microbial community functions. These changes exhibited both species-specific and time-dependent characteristics. *E. tenella* infection led to a significant increase in the abundance of environmental information processing pathways in the jejunum and a significant decrease in metabolism-related pathways in the cecum at 4 dpi, indicating a disruption of normal intestinal ecological balance and significant inhibition of basic metabolic activities in cecal microbiota. By 10 dpi, the abundance of genetic information processing in the cecum significantly decreased, while the abundance of environmental information processing pathways significantly increased. This indicated that cecal microbial communities had adapted functionally, shifting from typical genetic information processing and metabolic activities to a stress response mode, thereby actively perceiving and responding to intestinal disturbances caused by parasites. *E. maxima* infection caused a significant decrease in the abundance of metabolism-related pathway in the jejunum and genetic information processing-related pathway in the cecum. This is consistent with the findings reported by Su et al. [121], who demonstrated that *E. maxima* infection disrupts metabolic functions in chickens, particularly affecting nutrient absorption and energy metabolism pathways. *E. necatrix* infection also affected the intestinal microbial community functions of chickens, although these changes were not significantly different from those observed in the control group. This result might be related to the change in parasitic sites during the development of *E. necatrix* and the extent of intestinal damage. Resent research revealed that the severity of intestinal dysfunction caused by *E. necatrix* infection is dose-dependent [51].

## 5. Conclusions

This study demonstrates both coccidian species-specific and common changes in the gut microbiota of chickens infected with *E. tenella*, *E. maxima*, and *E. necatrix*. Specifically, all three *Eimeria* infections lead to a reduction in gut commensal bacteria, especially lactic acid bacteria. *Limosilactobacillus reuteri* consistently shows the greatest decline across all infections, suggesting its significance in maintaining intestinal microbiome balance and warranting further investigation into its potential anticoccidial properties. *E. tenella* infection causes an imbalance in the core microbiota characterized by the proliferation of opportunistic pathogens and invasion by exogenous bacteria, leading to acute inflammation and barrier disruption. *E. maxima* infection causes an imbalance in the core microbiota characterized by the proliferation of pathogenic bacteria and compensatory growth of anti-inflammatory bacteria, leading to pseudomembranous colitis and mucus layer loss. *E. necatrix* infection causes an imbalance in the core microbiota characterized by the activation of opportunistic pathogens and metabolic bacterial disorders, leading to infectious diarrhea and metabolic imbalance. These structural changes in microbiota were accompanied by corresponding functional alterations, with each *Eimeria* species causing unique disruptions in metabolic pathways. These findings provide crucial theoretical basis for understanding the pathogenic mechanisms of *Eimeria* and developing strategies for controlling coccidiosis through microecological regulation.

## Data Availability

The 16S rDNA sequencing raw data have been saved in the China National Center for Bioinformation, with the accession codes PRJCA030959 and PRJCA037815. All data and additional files are available from the corresponding author on reasonable request.

## Supplementary Materials

The following supporting information can be downloaded at https://www.mdpi.com/article/10.3390/microorganisms13122752/s1: Figure S1: The results of microscopic examination for parasites in intestinal mucosa at 4 and 10 days post-infection (dpi) with *E. tenella* (A–D), *E. maxima* (E–H), and *E. necatrix* (I–L) (400x magnification). (A) *E. tenella*, 4 dpi, jejunum—no parasites observed. (B) *E. tenella*, 4 dpi, cecum—numerous second-generation schizonts observed (50.1 µm × 43.4 µm). (C) *E. tenella*, 10 dpi, jejunum—no parasites observed. (D) *E. tenella*, 10 dpi, cecum—oocysts observed (22.3 µm × 19.5 µm). (E) *E. maxima*, 4 dpi, jejunum—developing gametocytes observed (12.4 µm × 11.5 µm). (F) *E. maxima*, 4 dpi, cecum—no parasites observed. (G) *E. maxima*, 10 dpi, jejunum—oocysts observed (28.0 µm × 19.8 µm). (H) *E. maxima*, 10 dpi, cecum—no parasites observed. (I) *E. necatrix*, 4 dpi, jejunum—second-generation schizonts observed (73.4 µm × 70.9 µm). (J) *E. necatrix*, 4 dpi, cecum—no parasites observed. (K) *E. necatrix*, 10 dpi, jejunum—numerous second-generation schizonts observed (54.2 µm × 41.2 µm). (L) *E. necatrix*, 10 dpi, cecum—oocysts observed (20.5 µm × 16.6 µm). Red arrows indicate parasites at different developmental stages and oocysts. Red arrows indicate the parasites; Figure S2: Curves of OTUs obtained from 80 samples. (A) Rarefaction curves. (B) Shannon-Wiener curves; Table S1: Group information of 80 fecal samples in this study; Table S2: Summary of sequenced samples; Table S3: Abundance of 130,962 bacterial OTUs after flattening and species annotation; Table S4: Alpha index statistics; Table S5: The number of samples at various taxonomic levels.

## References

[1] Blake D.P., Knox J., Dehaeck B., Huntington B., Rathinam T., Ravipati V., Ayoade S., Gilbert W., Adebambo A.O., Jatau I.D. (2020). Re-calculating the cost of coccidiosis in chickens. Vet. Res..

[2] Lee Y., Lu M., Lillehoj H.S. (2022). Coccidiosis: Recent Progress in Host Immunity and Alternatives to Antibiotic Strategies. Vaccines.

[3] Peek H.W., Landman W.J.M. (2011). Coccidiosis in poultry: Anticoccidial products, vaccines and other prevention strategies. Vet. Q..

[4] Shang Y., Kumar S., Oakley B., Kim W.K. (2018). Chicken Gut Microbiota: Importance and Detection Technology. Front. Vet. Sci..

[5] Stanley D., Hughes R.J., Moore R.J. (2014). Microbiota of the chicken gastrointestinal tract: Influence on health, productivity and disease. Appl. Microbiol. Biotechnol..

[6] Clavijo V., Flórez M.J.V. (2018). The gastrointestinal microbiome and its association with the control of pathogens in broiler chicken production: A review. Poult. Sci..

[7] Huang G., Zhang S., Zhou C., Tang X., Li C., Wang C., Tang X., Suo J., Jia Y., El-Ashram S. (2018). Influence of *Eimeria falciformis* Infection on Gut Microbiota and Metabolic Pathways in Mice. Infect. Immun..

[8] Madlala T., Okpeku M., Adeleke M.A. (2021). Understanding the interactions between *Eimeria* infection and gut microbiota, towards the control of chicken coccidiosis: A review. Parasite.

[9] Antonissen G., Eeckhaut V., Van Driessche K., Onrust L., Haesebrouck F., Ducatelle R., Moore R.J., Van Immerseel F. (2016). Microbial shifts associated with necrotic enteritis. Avian Pathol. J. WVPA.

[10] Hauck R. (2017). Interactions Between Parasites and the Bacterial Microbiota of Chickens. Avian Dis..

[11] Macdonald S.E., van Diemen P.M., Martineau H., Stevens M.P., Tomley F.M., Stabler R.A., Blake D.P. (2019). Impact of *Eimeria tenella* Coinfection on Campylobacter jejuni Colonization of the Chicken. Infect. Immun..

[12] Gadde U., Kim W.H., Oh S.T., Lillehoj H.S. (2017). Alternatives to antibiotics for maximizing growth performance and feed efficiency in poultry: A review. Anim. Health Res. Rev..

[13] Chen H.-L., Zhao X.-Y., Zhao G.-X., Huang H.-B., Li H.-R., Shi C.-W., Yang W.-T., Jiang Y.-L., Wang J.-Z., Ye L.-P. (2020). Dissection of the cecal microbial community in chickens after *Eimeria tenella* infection. Parasites Vectors.

[14] Sun L., Liu Y., Xiao P., Zhang K., Bai S., Wang J., Zeng Q., Peng H., Mu Y., Xuan Y. (2024). Probiotic *Bacillus subtilis* QST713 improved growth performance and enhanced the intestinal health of yellow-feather broilers challenged with coccidia and *Clostridium perfringens*. Poult. Sci..

[15] Song X., Gao Y., Xu L., Yan R., Li X. (2015). Partial protection against four species of chicken coccidia induced by multivalent subunit vaccine. Vet. Parasitol..

[16] Lan L.H., Sun B.B., Zuo B.X.Z., Chen X.Q., Du A.F. (2017). Prevalence and drug resistance of avian *Eimeria* species in broiler chicken farms of Zhejiang province, China. Poult. Sci..

[17] Chengat Prakashbabu B., Thenmozhi V., Limon G., Kundu K., Kumar S., Garg R., Clark E., Rao A.S., Raj D., Raman M. (2016). *Eimeria* species occurrence varies between geographic regions and poultry production systems and may influence parasite genetic diversity. Vet. Parasitol..

[18] Rose M.E., Hesketh P. (1976). Immunity to coccidiosis: Stages of the life-cycle of *Eimeria maxima* which induce, and are affected by, the response of the host. Parasitology.

[19] McDougald L.R., Cervantes H.M., Jenkins M.C., Hess M., Beckstead R. (2020). Protozoal Infections. Diseases of Poultry.

[20] Huang G., Tang X., Bi F., Hao Z., Han Z., Suo J., Zhang S., Wang S., Duan C., Yu Z. (2018). *Eimeria tenella* infection perturbs the chicken gut microbiota from the onset of oocyst shedding. Vet. Parasitol..

[21] McDonald V., Shirley M.W. (1987). The endogenous development of virulent strains and attenuated precocious lines of *Eimeria tenella* and *E. necatrix*. J. Parasitol..

[22] Zhou B.H., Jia L.S., Wei S.S., Ding H.Y., Yang J.Y., Wang H.W. (2020). Effects of *Eimeria tenella* infection on the barrier damage and microbiota diversity of chicken cecum. Poult. Sci..

[23] Choi J., Kim W. (2022). Interactions of Microbiota and Mucosal Immunity in the Ceca of Broiler Chickens Infected with *Eimeria tenella*. Vaccines.

[24] Lu M., Li R.W., Zhao H., Yan X., Lillehoj H.S., Sun Z., Oh S., Wang Y., Li C. (2020). Effects of *Eimeria maxima* and *Clostridium perfringens* infections on cecal microbial composition and the possible correlation with body weight gain in broiler chickens. Res. Vet. Sci..

[25] Gao Y., Suding Z., Wang L., Liu D., Su S., Xu J., Hu J., Tao J. (2021). Full-length transcriptome analysis and identification of transcript structures in *Eimeria necatrix* from different developmental stages by single-molecule real-time sequencing. Parasites Vectors.

[26] Liu D., Wang F., Cao L., Wang L., Su S., Hou Z., Xu J., Hu J., Tao J. (2020). Identification and characterization of a cDNA encoding a gametocyte-specific protein of the avian coccidial parasite *Eimeria necatrix*. Mol. Biochem. Parasitol..

[27] Xue N., Feng Q., Zhu Y., Cheng C., Wang F., Liu D., Su S., Xu J., Hu J., Tao J. (2025). Full-length 16S rRNA sequencing revealed an altered microbiome diversity and composition of the jejunum and cecum in chicken infected with *Eimeria necatrix*. Vet. Parasitol..

[28] Kaingu F., Liu D., Wang L., Tao J., Waihenya R., Kutima H. (2017). Anticoccidial effects of Aloe secundiflora leaf extract against *Eimeria tenella* in broiler chicken. Trop. Anim. Health Prod..

[29] Liu D., Cao L., Zhu Y., Deng C., Su S., Xu J., Jin W., Li J., Wu L., Tao J. (2014). Cloning and characterization of an *Eimeria necatrix* gene encoding a gametocyte protein and associated with oocyst wall formation. Parasites Vectors.

[30] Liu D., Li J., Cao L., Wang S., Han H., Wu Y., Tao J. (2014). Analysis of differentially expressed genes in two immunologically distinct strains of *Eimeria maxima* using suppression subtractive hybridization and dot-blot hybridization. Parasites Vectors.

[31] Quast C., Pruesse E., Yilmaz P., Gerken J., Schweer T., Yarza P., Peplies J., Glöckner F.O. (2013). The SILVA ribosomal RNA gene database project: Improved data processing and web-based tools. Nucleic Acids Res..

[32] Schloss P.D., Westcott S.L., Ryabin T., Hall J.R., Hartmann M., Hollister E.B., Lesniewski R.A., Oakley B.B., Parks D.H., Robinson C.J. (2009). Introducing mothur: Open-source, platform-independent, community-supported software for describing and comparing microbial communities. Appl. Environ. Microbiol..

[33] Oksanen J., Simpson G., Blanchet F.G., Kindt R., Legendre P., Minchin P., O’hara R.B., Solymos P., Stevens M.H.H., Szoecs E. (2022). Vegan Community Ecology Package. Version 2.6-2. https://CRAN.R-project.org/package=vegan.

[34] Lozupone C., Lladser M.E., Knights D., Stombaugh J., Knight R. (2011). UniFrac: An effective distance metric for microbial community comparison. ISME J..

[35] Segata N., Izard J., Waldron L., Gevers D., Miropolsky L., Garrett W.S., Huttenhower C. (2011). Metagenomic biomarker discovery and explanation. Genome Biol..

[36] Langille M.G.I., Zaneveld J., Caporaso J.G., McDonald D., Knights D., Reyes J.A., Clemente J.C., Burkepile D.E., Vega Thurber R.L., Knight R. (2013). Predictive functional profiling of microbial communities using 16S rRNA marker gene sequences. Nat. Biotechnol..

[37] Dittoe D.K., Olson E.G., Ricke S.C. (2022). Impact of the gastrointestinal microbiome and fermentation metabolites on broiler performance. Poult. Sci..

[38] Ncho C.M. (2025). Heat stress and the chicken gastrointestinal microbiota: A systematic review. J. Anim. Sci. Biotechnol..

[39] Mesa-Pineda C., Navarro-Ruíz J.L., López-Osorio S., Chaparro-Gutiérrez J.J., Gómez-Osorio L.M. (2021). Chicken Coccidiosis: From the Parasite Lifecycle to Control of the Disease. Front. Vet. Sci..

[40] Mathis G.F., Lumpkins B., Cervantes H.M., Fitz-Coy S.H., Jenkins M.C., Jones M.K., Price K.R., Dalloul R.A. (2025). Coccidiosis in poultry: Disease mechanisms, control strategies, and future directions. Poult. Sci..

[41] Qiu M., Hu J., Peng H., Li B., Xu J., Song X., Yu C., Zhang Z., Du X., Bu G. (2022). Research Note: The gut microbiota varies with dietary fiber levels in broilers. Poult. Sci..

[42] Bindari Y.R., Gerber P.F. (2022). Centennial Review: Factors affecting the chicken gastrointestinal microbial composition and their association with gut health and productive performance. Poult. Sci..

[43] Burrows P.B., Godoy-Santos F., Lawther K., Richmond A., Corcionivoschi N., Huws S.A. (2025). Decoding the chicken gastrointestinal microbiome. BMC Microbiol..

[44] Chica Cardenas L.A., Clavijo V., Vives M., Reyes A. (2021). Bacterial meta-analysis of chicken cecal microbiota. PeerJ.

[45] Wexler H.M. (2007). Bacteroides: The good, the bad, and the nitty-gritty. Clin. Microbiol. Rev..

[46] Fan Y., Ju T., Bhardwaj T., Korver D.R., Willing B.P. (2023). Week-Old Chicks with High *Bacteroides* Abundance Have Increased Short-Chain Fatty Acids and Reduced Markers of Gut Inflammation. Microbiol. Spectr..

[47] Mamieva Z., Poluektova E., Svistushkin V., Sobolev V., Shifrin O., Guarner F., Ivashkin V. (2022). Antibiotics, gut microbiota, and irritable bowel syndrome: What are the relations?. World J. Gastroenterol..

[48] Rizzatti G., Lopetuso L.R., Gibiino G., Binda C., Gasbarrini A. (2017). Proteobacteria: A Common Factor in Human Diseases. BioMed Res. Int..

[49] Yu X., Niu S., Tie K., Zhang Q., Deng H., Gao C., Yu T., Lei L., Feng X. (2019). Characteristics of the intestinal flora of specific pathogen free chickens with age. Microb. Pathog..

[50] Du S., Song Z., Cen Y., Fan J., Li P., Si H., Hu D. (2024). Susceptibility and cecal microbiota alteration to *Eimeria*-infection in Yellow-feathered broilers, Arbor Acres broilers and Lohmann pink layers. Poult. Sci..

[51] Chen Y.M., Wei P., Liao H.Y., Tsai Y.W., Cheng M.C., Lien Y.Y. (2025). Comprehensive analysis of *Eimeria necatrix* infection: From intestinal lesions to gut microbiota and metabolic disturbances. Poult. Sci..

[52] Liu J., Guo J., Whitmore M.A., Tobin I., Kim D.M., Zhao Z., Zhang G. (2024). Dynamic response of the intestinal microbiome to *Eimeria maxima*-induced coccidiosis in chickens. Microbiol. Spectr..

[53] Kim S., Covington A., Pamer E.G. (2017). The intestinal microbiota: Antibiotics, colonization resistance, and enteric pathogens. Immunol. Rev..

[54] Macdonald S.E., Nolan M.J., Harman K., Boulton K., Hume D.A., Tomley F.M., Stabler R.A., Blake D.P. (2017). Effects of *Eimeria tenella* infection on chicken caecal microbiome diversity, exploring variation associated with severity of pathology. PLoS ONE.

[55] Zhou Z., Nie K., Huang Q., Li K., Sun Y., Zhou R., Wang Z., Hu S. (2017). Changes of cecal microflora in chickens following Eimeria tenella challenge and regulating effect of coated sodium butyrate. Exp. Parasitol..

[56] Jebessa E., Guo L., Chen X., Bello S.F., Cai B., Girma M., Hanotte O., Nie Q. (2022). Influence of *Eimeria maxima* coccidia infection on gut microbiome diversity and composition of the jejunum and cecum of indigenous chicken. Front. Immunol..

[57] Yu H., Wang Q., Tang J., Dong L., Dai G., Zhang T., Zhang G., Xie K., Wang H., Zhao Z. (2023). Comprehensive analysis of gut microbiome and host transcriptome in chickens after *Eimeria tenella* infection. Front. Cell. Infect. Microbiol..

[58] Zheng J., Wittouck S., Salvetti E., Franz C.M.A.P., Harris H.M.B., Mattarelli P., O’Toole P.W., Pot B., Vandamme P., Walter J. (2020). A taxonomic note on the genus *Lactobacillus*: Description of 23 novel genera, emended description of the genus *Lactobacillus Beijerinck* 1901, and union of Lactobacillaceae and Leuconostocaceae. Int. J. Syst. Evol. Microbiol..

[59] Li F., Cheng C.C., Zheng J., Liu J., Quevedo R.M., Li J., Roos S., Gänzle M.G., Walter J. (2021). *Limosilactobacillus balticus* sp. nov., *Limosilactobacillus agrestis* sp. nov., *Limosilactobacillus albertensis* sp. nov., *Limosilactobacillus rudii* sp. nov. and *Limosilactobacillus fastidiosus* sp. nov., five novel *Limosilactobacillus* species isolated from the vertebrate gastrointestinal tract, and proposal of six subspecies of *Limosilactobacillus reuteri* adapted to the gastrointestinal tract of specific vertebrate hosts. Int. J. Syst. Evol. Microbiol..

[60] Nii T., Kakuya H., Isobe N., Yoshimura Y. (2020). *Lactobacillus reuteri* Enhances the Mucosal Barrier Function Against Heat-killed Salmonella Typhimurium in the Intestine of Broiler Chicks. J. Poult. Sci..

[61] Chai C., Guo Y., Mohamed T., Bumbie G.Z., Wang Y., Zeng X., Zhao J., Du H., Tang Z., Xu Y. (2023). Dietary *Lactobacillus reuteri* SL001 Improves Growth Performance, Health-Related Parameters, Intestinal Morphology and Microbiota of Broiler Chickens. Animals.

[62] He T., Hu X., Mi J., Hu H., Wang H., Qi X., Gao L., Zhang Y., Liu C., Wang S. (2024). *Ligilactobacillus salivarius* XP132 with antibacterial and immunomodulatory activities inhibits horizontal and vertical transmission of Salmonella Pullorum in chickens. Poult. Sci..

[63] Eeckhaut V., Van Immerseel F., Croubels S., De Baere S., Haesebrouck F., Ducatelle R., Louis P., Vandamme P. (2011). Butyrate production in phylogenetically diverse Firmicutes isolated from the chicken caecum. Microb. Biotechnol..

[64] Zhang J., Zhang H., Wang L., Zhang K., Qiu Z., Zhang K., Yue C., Zhang Y., Zhao X., Li J. (2021). The safety and potential probiotic properties analysis of *Streptococcus alactolyticus* strain FGM isolated from the chicken cecum. Ann. Microbiol..

[65] Ribeiro J., Silva V., Monteiro A., Vieira-Pinto M., Igrejas G., Reis F.S., Barros L., Poeta P. (2023). Antibiotic Resistance among Gastrointestinal Bacteria in Broilers: A Review Focused on *Enterococcus* spp. and *Escherichia coli*. Animals.

[66] Parker B.J., Wearsch P.A., Veloo A.C.M., Rodriguez-Palacios A. (2020). The Genus *Alistipes*: Gut Bacteria with Emerging Implications to Inflammation, Cancer, and Mental Health. Front. Immunol..

[67] Kralova S., Davidova-Gerzova L., Valcek A., Bezdicek M., Rychlik I., Rezacova V., Cizek A. (2022). *Paraphocaeicola brunensis* gen. nov., sp. nov., Carrying Two Variants of nimB Resistance Gene from *Bacteroides fragilis,* and *Caecibacteroides pullorum* gen. nov., sp. nov., Two Novel Genera Isolated from Chicken Caeca. Microbiol. Spectr..

[68] Wexler A.G., Goodman A.L. (2017). An insider’s perspective: *Bacteroides* as a window into the microbiome. Nat. Microbiol..

[69] Mai X., Yang S., Chen Q., Chen K. (2024). Gut microbial composition is altered in sarcopenia: A systematic review and meta-analysis of clinical studies. PLoS ONE.

[70] Di Marcantonio L., Marotta F., Vulpiani M.P., Sonntag Q., Iannetti L., Janowicz A., Di Serafino G., Di Giannatale E., Garofolo G. (2022). Investigating the cecal microbiota in broiler poultry farms and its potential relationships with animal welfare. Res. Vet. Sci..

[71] Niu Q., Wang X., Qi X., Cao C., Yang K., Gu C., Zhou Z., Huang Q. (2023). Identification of the gut microbiota affecting *Salmonella pullorum* and their relationship with reproductive performance in hens. Front. Microbiol..

[72] Xiao X., Cui T., Qin S., Wang T., Liu J., Sa L., Wu Y., Zhong Y., Yang C. (2024). Beneficial effects of *Lactobacillus plantarum* on growth performance, immune status, antioxidant function and intestinal microbiota in broilers. Poult. Sci..

[73] Chen X., Bai H., Mo W., Zheng X., Chen H., Yin Y., Liao Y., Chen Z., Shi Q., Zuo Z. (2025). Lactic Acid Bacteria Bacteriocins: Safe and Effective Antimicrobial Agents. Int. J. Mol. Sci..

[74] Lee A.H., Rodriguez Jimenez D.M., Meisel M. (2025). *Limosilactobacillus reuteri*—A probiotic gut commensal with contextual impact on immunity. Gut Microbes.

[75] Hou K., Wu Z.X., Chen X.Y., Wang J.Q., Zhang D., Xiao C., Zhu D., Koya J.B., Wei L., Li J. (2022). Microbiota in health and diseases. Signal Transduct. Target. Ther..

[76] Mohd Shaufi M.A., Sieo C.C., Chong C.W., Gan H.M., Ho Y.W. (2015). Deciphering chicken gut microbial dynamics based on high-throughput 16S rRNA metagenomics analyses. Gut Pathog..

[77] Jiang T., Liu K., Zhang Y., Zhang W., Doherty M., Yang T., Li J., Weng Q., Wei J., Zeng C. (2023). Gut Fungal Dysbiosis and Altered Fungi-Bacteria Correlation Network Were Associated with Knee Synovitis in a Middle-Aged and Older General Population in China. Osteoarthr. Cartil..

[78] Morotomi M., Nagai F., Watanabe Y. (2012). Description of *Christensenella minuta* gen. nov., sp. nov., isolated from human faeces, which forms a distinct branch in the order Clostridiales, and proposal of *Christensenellaceae fam*. nov. Int. J. Syst. Evol. Microbiol..

[79] Ignatyeva O., Tolyneva D., Kovalyov A., Matkava L., Terekhov M., Kashtanova D., Zagainova A., Ivanov M., Yudin V., Makarov V. (2023). *Christensenella minuta*, a new candidate next-generation probiotic: Current evidence and future trajectories. Front. Microbiol..

[80] Kassab N.S., Alsammarraie N., Sarsam T., Al-Sammarraie M., Watt J. (2024). A Rare Case of *Gemella haemolysans* Endocarditis: A Challenging Diagnosis. Cureus.

[81] Purohit G., Mishra B., Sahoo S., Mahapatra A. (2022). *Granulicatella adiacens* as an Unusual Cause of Empyema: A Case Report and Review of Literature. J. Lab. Physicians.

[82] Kodaka S., Uchida T., Gomi H. (2022). *Gemella haemolysans* as an emerging pathogen for bacteremia among the elderly. J. Gen. Fam. Med..

[83] Mandal R.K., Jiang T., Al-Rubaye A.A., Rhoads D.D., Wideman R.F., Zhao J., Pevzner I., Kwon Y.M. (2016). An investigation into blood microbiota and its potential association with Bacterial Chondronecrosis with Osteomyelitis (BCO) in Broilers. Sci. Rep..

[84] Haas K.N., Blanchard J.L. (2020). Reclassification of the *Clostridium clostridioforme* and *Clostridium sphenoides* clades as *Enterocloster* gen. nov. and *Lacrimispora* gen. nov., including reclassification of 15 taxa. Int. J. Syst. Evol. Microbiol..

[85] Beresford-Jones B.S., Suyama S., Clare S., Soderholm A., Xia W., Sardar P., Lee J., Harcourt K., Lawley T.D., Pedicord V.A. (2025). *Enterocloster clostridioformis* protects against *Salmonella* pathogenesis and modulates epithelial and mucosal immune function. Microbiome.

[86] Cobo F., Rodríguez-Granger J., Navarro-Mari J.M., Ceballos-Atienza R., Sampedro-Martínez A., Reguera-Márquez J.A. (2025). Four cases of bacteremia caused by *Enterocloster clostridioformis*. Rev. Esp. Quimioter. Publ. Of. Soc. Esp. Quimioter..

[87] Andrieu C., Mailhe M., Ricaboni D., Fonkou M.D.M., Bilen M., Cadoret F., Tomei E., Armstrong N., Vitton V., Benezech A. (2018). Noncontiguous finished genome sequences and description of *Bacteroides mediterraneensis* sp. nov., *Bacteroides ihuae* sp. nov., *Bacteroides togonis* sp. nov., *Bacteroides ndongoniae* sp. nov., *Bacteroides ilei* sp. nov. and *Bacteroides Congonensis* sp. nov. Identified by culturomics. New Microbes New Infect..

[88] Sakamoto M., Lan P.T.N., Benno Y. (2007). *Barnesiella viscericola* gen. nov., sp. nov., a novel member of the family Porphyromonadaceae isolated from chicken caecum. Int. J. Syst. Evol. Microbiol..

[89] Medina Fernández S., Cretenet M., Bernardeau M. (2019). In vitro inhibition of avian pathogenic *Enterococcus cecorum* isolates by probiotic Bacillus strains. Poult. Sci..

[90] Kim B., Kim M., Lee K., Lee Y. (2025). Clinical Outcomes and Molecular Characteristics of *Bacteroides fragilis* Infections. Ann. Lab. Med..

[91] Wrigley D.M. (2004). Inhibition of Clostridium perfringens sporulation by *Bacteroides fragilis* and short-chain fatty acids. Anaerobe.

[92] Chen L., Yang M., Zhu W., Su Y., Li D., Wang T. (2022). Multi-Omics Analysis After Vaginal Administration of *Bacteroides fragilis* in Chickens. Front. Microbiol..

[93] Zhang S., You M., Shen Y., Zhao X., He X., Liu J., Ma N. (2025). Improving fatty liver hemorrhagic syndrome in laying hens through gut microbiota and oxylipin metabolism by *Bacteroides fragilis*: A potential involvement of arachidonic acid. Anim. Nutr. (Zhongguo Xu Mu Shou Yi Xue Hui).

[94] Gu H. (2017). Role of Flagella in the Pathogenesis of *Helicobacter pylori*. Curr. Microbiol..

[95] Hamada M., Elbehiry A., Marzouk E., Moussa I.M., Hessain A.M., Alhaji J.H., Heme H.A., Zahran R., Abdeen E. (2018). *Helicobacter pylori* in a poultry slaughterhouse: Prevalence, genotyping and antibiotic resistance pattern. Saudi J. Biol. Sci..

[96] Vorob’ev A.V., de Boer W., Folman L.B., Bodelier P.L.E., Doronina N.V., Suzina N.E., Trotsenko Y.A., Dedysh S.N. (2009). *Methylovirgula ligni* gen. nov., sp. nov., an obligately acidophilic, facultatively methylotrophic bacterium with a highly divergent mxaF gene. Int. J. Syst. Evol. Microbiol..

[97] Kuehne S.A., Cartman S.T., Heap J.T., Kelly M.L., Cockayne A., Minton N.P. (2010). The role of toxin A and toxin B in *Clostridium difficile* infection. Nature.

[98] Salas-Treviño D., Flores-Treviño S., Cisneros-Rendón C., Domínguez-Rivera C.V., Camacho-Ortiz A. (2025). Co-Colonization of Non-difficile Clostridial Species in Antibiotic-Associated Diarrhea Caused by *Clostridioides difficile*. Antibiotics.

[99] Weese J.S. (2020). *Clostridium* (Clostridioides) difficile in animals. J. Vet. Diagn. Investig..

[100] Sokol H., Pigneur B., Watterlot L., Lakhdari O., Bermúdez-Humarán L.G., Gratadoux J.-J., Blugeon S., Bridonneau C., Furet J.-P., Corthier G. (2008). *Faecalibacterium prausnitzii* is an anti-inflammatory commensal bacterium identified by gut microbiota analysis of Crohn disease patients. Proc. Natl. Acad. Sci. USA.

[101] Lenoir M., Martín R., Torres-Maravilla E., Chadi S., González-Dávila P., Sokol H., Langella P., Chain F., Bermúdez-Humarán L.G. (2020). Butyrate mediates anti-inflammatory effects of *Faecalibacterium prausnitzii* in intestinal epithelial cells through Dact3. Gut Microbes.

[102] Wongkuna S., Ghimire S., Chankhamhaengdecha S., Janvilisri T., Scaria J. (2021). *Mediterraneibacter catenae* SW178 sp. nov., an intestinal bacterium of feral chicken. PeerJ.

[103] Glover J.S., Ticer T.D., Engevik M.A. (2022). Characterizing the mucin-degrading capacity of the human gut microbiota. Sci. Rep..

[104] Zhou Y., Zhao X., Zhang M., Feng J. (2022). Gut microbiota dysbiosis exaggerates ammonia-induced tracheal injury Via TLR4 signaling pathway. Ecotoxicol. Environ. Saf..

[105] Bang S., Shin Y.H., Ma X., Park S.M., Graham D.B., Xavier R.J., Clardy J. (2023). A Cardiolipin from *Muribaculum intestinale* Induces Antigen-Specific Cytokine Responses. J. Am. Chem. Soc..

[106] Wang Z., Kang S., Wu Z., Liu X., Zhang X., Wu Y., Wen Y., Zhou X., Zhang G., Wang J. (2025). *Muribaculum intestinale* restricts *Salmonella Typhimurium* colonization by converting succinate to propionate. ISME J..

[107] Liébana-García R., López-Almela I., Olivares M., Romaní-Pérez M., Manghi P., Torres-Mayo A., Tolosa-Enguís V., Flor-Duro A., Bullich-Vilarrubias C., Rubio T. (2025). Gut commensal *Phascolarctobacterium faecium* retunes innate immunity to mitigate obesity and metabolic disease in mice. Nat. Microbiol..

[108] García-López M., Meier-Kolthoff J.P., Tindall B.J., Gronow S., Woyke T., Kyrpides N.C., Hahnke R.L., Göker M. (2019). Analysis of 1000 Type-Strain Genomes Improves Taxonomic Classification of Bacteroidetes. Front. Microbiol..

[109] Chen H.L., Hu P.Y., Chen C.S., Lin W.H., Hsu D.K., Liu F.T., Meng T.-C. (2025). Gut colonization of *Bacteroides plebeius* suppresses colitis-associated colon cancer development. Microbiol. Spectr..

[110] Thiam M., Wang Q., Barreto Sánchez A.L., Zhang J., Ding J., Wang H., Zhang Q., Zhang N., Wang J., Li Q. (2022). Heterophil/Lymphocyte Ratio Level Modulates *Salmonella* Resistance, Cecal Microbiota Composition and Functional Capacity in Infected Chicken. Front. Immunol..

[111] Khanom H., Nath C., Mshelbwala P.P., Pasha M.R., Magalhaes R.S., Alawneh J.I., Hassan M.M. (2025). Epidemiology and molecular characterisation of multidrug-resistant *Escherichia coli* isolated from chicken meat. PLoS ONE.

[112] Schwarzerova J., Zeman M., Babak V., Jureckova K., Nykrynova M., Varga M., Weckwerth W., Dolejska M., Provaznik V., Rychlik I. (2024). Detecting horizontal gene transfer among microbiota: An innovative pipeline for identifying co-shared genes within the mobilome through advanced comparative analysis. Microbiol. Spectr..

[113] Choi S.H., Kim J.S., Park J.E., Lee K.C., Eom M.K., Oh B.S., Yu S.Y., Kang S.W., Han K.-I., Suh M.K. (2019). *Anaerotignum faecicola* sp. nov., isolated from human faeces. J. Microbiol..

[114] Ueki A., Goto K., Ohtaki Y., Kaku N., Ueki K. (2017). Description of *Anaerotignum aminivorans* gen. nov., sp. nov., a strictly anaerobic, amino-acid-decomposing bacterium isolated from a methanogenic reactor, and reclassification of *Clostridium propionicum*, *Clostridium neopropionicum* and *Clostridium lactatifermentans* as species of the genus *Anaerotignum*. Int. J. Syst. Evol. Microbiol..

[115] Zhu J., Li X., Deng N., Peng X., Tan Z. (2022). Diarrhea with deficiency kidney-yang syndrome caused by adenine combined with Folium senna was associated with gut mucosal microbiota. Front. Microbiol..

[116] Petzoldt D., Breves G., Rautenschlein S., Taras D. (2016). *Harryflintia acetispora* gen. nov., sp. nov., isolated from chicken caecum. Int. J. Syst. Evol. Microbiol..

[117] Drent W.J., Lahpor G.A., Wiegant W.M., Gottschal J.C. (1991). Fermentation of Inulin by *Clostridium thermosuccinogenes* sp. nov., a Thermophilic Anaerobic Bacterium Isolated from Various Habitats. Appl. Environ. Microbiol..

[118] Koendjbiharie J.G., Wiersma K., van Kranenburg R. (2018). Investigating the Central Metabolism of *Clostridium thermosuccinogenes*. Appl. Environ. Microbiol..

[119] Mailhe M., Ricaboni D., Benezech A., Lagier J.C., Fournier P.E., Raoult D. (2017). ‘*Tidjanibacter massiliensis*’ gen. nov., sp. nov., a new bacterial species isolated from human colon. New Microbes New Infect..

[120] Du L., Chen W., Wang J., Huang L., Zheng Q., Chen J., Wang L., Cai C., Zhang X., Wang L. (2023). Beneficial Effects of *Bacillus amyloliquefaciens* D1 Soy Milk Supplementation on Serum Biochemical Indexes and Intestinal Health of Bearded Chickens. Microorganisms.

[121] Su S., Yang J., Xu L., Su D., Liu D., Xu J., Tao J. (2025). Combined serum lipid levels and lipidomic analysis reveals effects of *Eimeria maxima* and *Eimeria tenella* infection on lipid metabolism in chicken. Vet. Parasitol..

