# Supplementary material for: Comparison of Gut Microbiome Profile of Chickens Infected with Three Eimeria Species Reveals New Insights on Pathogenicity of Avian Coccidia
Source: Microorganisms. 2025 Dec 3;13(12):2752. doi: 10.3390/microorganisms13122752 (PMC12736274; doi:10.3390/microorganisms13122752)
Supplement: Supplementary file 1 [file microorganisms-13-02752-s001.zip › microorganisms-3969231-supplementary.pdf]

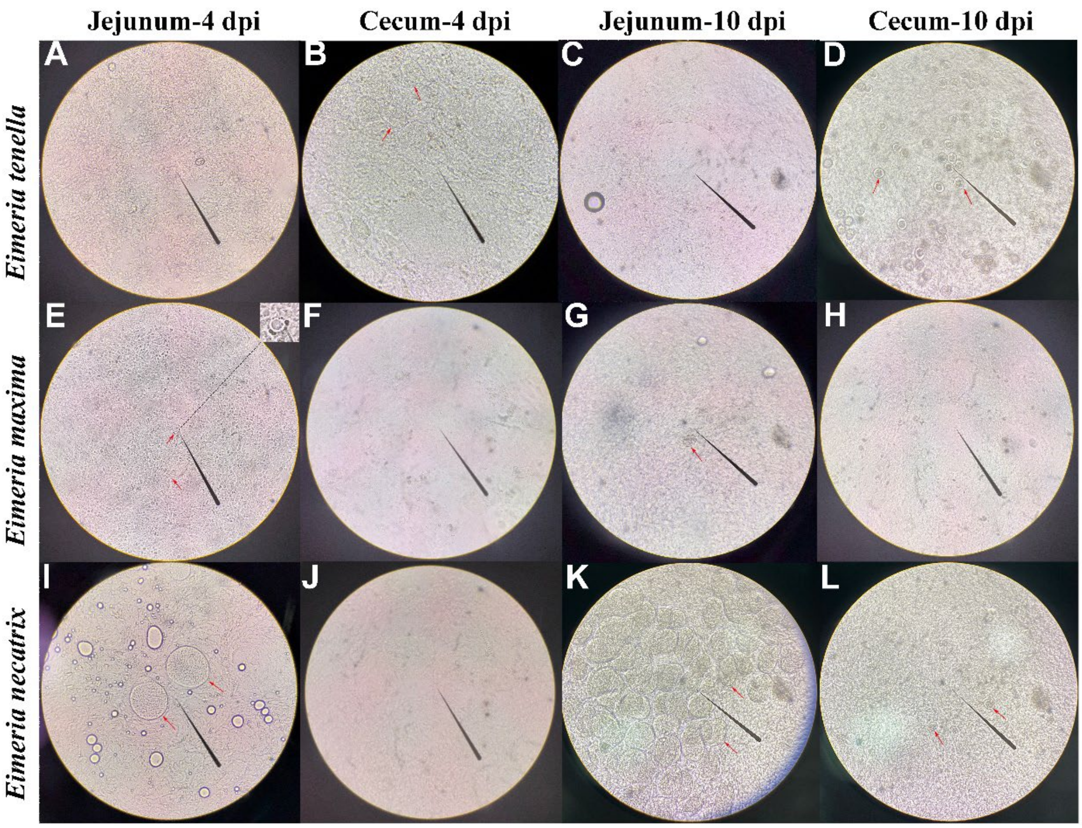

Figure S1: The results of microscopic examination for parasites in intestinal mucosa at 4 and 10 days post-infection (dpi) with *E. tenella* (A-D), *E. maxima* (E-H), and *E. necatrix* (I-L) (400x magnification). (A) *E. tenella*, 4 dpi, jejunum – no parasites observed. (B) *E. tenella*, 4 dpi, cecum – numerous second-generation schizonts observed. (C) *E. tenella*, 10 dpi, jejunum – no parasites observed. (D) *E. tenella*, 10 dpi, cecum – oocysts observed. (E) *E. maxima*, 4 dpi, jejunum – developing gametocytes observed. (F) *E. maxima*, 4 dpi, cecum – no parasites observed. (G) *E. maxima*, 10 dpi, jejunum – oocysts observed. (H) *E. maxima*, 10 dpi, cecum – no parasites observed. (I) *E. necatrix*, 4 dpi, jejunum – second-generation schizonts observed. (J) *E. necatrix*, 4 dpi, cecum – no parasites observed. (K) *E. necatrix*, 10 dpi, jejunum – numerous second-generation schizonts observed. (L) *E. necatrix*, 10 dpi, cecum – oocysts observed. Red arrows indicate parasites at different developmental stages and oocysts. Red arrows indicate the parasites.

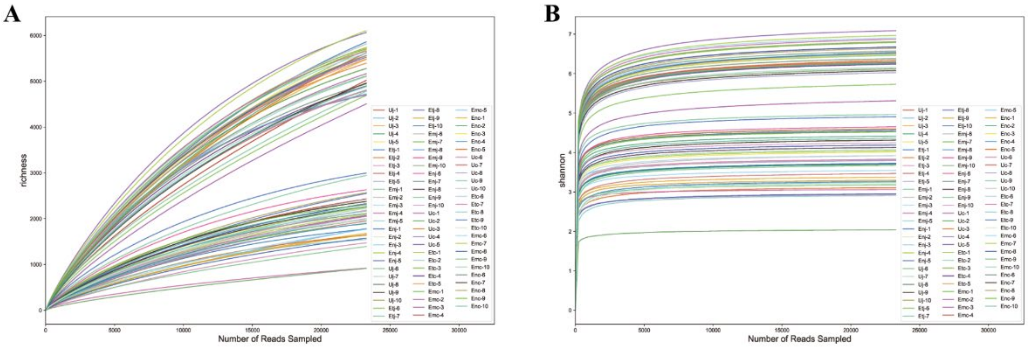

Figure S2: Curves of OTUs obtained from 80 samples. (A) Rarefaction curves. (B) Shannon-Wiener curves.
